# Scenario based outdoor simulation in pre-hospital trauma care using a simple mannequin model

**DOI:** 10.1186/1757-7241-18-13

**Published:** 2010-03-15

**Authors:** Per P Bredmose, Karel Habig, Gareth Davies, Gareth Grier, David J Lockey

**Affiliations:** 1 London Helicopter Emergency Medical Service, Department of Pre-hospital Care, The Royal London Hospital, London E1 1BB, UK

## Abstract

**Introduction:**

We describe a system of scenario-based training using simple mannequins under realistic circumstances for the training of pre-hospital care providers.

**Methods:**

A simple intubatable mannequin or student volunteers are used together with a training version of the equipment used on a routine basis by the pre-hospital care team (doctor + paramedic).

Training is conducted outdoors at the base location all year round. The scenarios are led by scenario facilitators who are predominantly senior physicians. Their role is to brief the training team and guide the scenario, results of patient assessment and the simulated responses to interventions and treatment. Pilots, fire-fighters and medical students are utilised in scenarios to enhance realism by taking up roles as bystanders, additional ambulance staff and police. These scenario participants are briefed and introduced to the scene in a realistic manner. After completion of the scenario, the training team would usually be invited to prepare and deliver a hospital handover as they would in a real mission. A formal structured debrief then takes place.

**Results:**

This training method technique has been used for the training of all London Helicopter Emergency Medical Service (London HEMS) doctors and paramedics over the last 24 months. Informal participant feedback suggests that this is a very useful teaching method, both for improving motor skills, critical decision-making, scene management and team interaction. Although formal assessment of this technique has not yet taken place we describe how this type of training is conducted in a busy operational pre-hospital trauma service.

**Discussion:**

The teaching and maintenance of pre-hospital care skills is essential to an effective pre-hospital trauma care system. Simple mannequin based scenario training is feasible on a day-to-day basis and has the advantages of low cost, rapid set up and turn around. The scope of scenarios is limited only by the imagination of the trainers. Significant effort is made to put the participants into "the Zone" - the psychological mindset, where they believe they are in a realistic setting and treating a real patient, so that they gain the most from each teaching session. The method can be used for learning new skills, communication and leadership as well as maintaining existing skills.

**Conclusion:**

The method described is a low technology, low cost alternative to high technology simulation which may provide a useful adjunct to delivering effective training when properly prepared and delivered. We find this useful for both induction and regular training of pre-hospital trauma care providers.

## Introduction

Delivering effective critical care to patients suffering major traumatic injury in the pre-hospital environment is highly demanding. Clinicians must be able to rapidly assess both the "scene" and their patients, utilize a variety of critical interventions and be able to operate effectively in stressful and sometimes hazardous environments. To ensure the highest possible standard of care clinicians must develop a large number of skills and competencies and practise them frequently to maintain clinical efficacy. To achieve these aims London Helicopter Emergency Medical Service (London HEMS) is involved in extensive training and assessment of doctors and paramedics in pre-hospital trauma care. "Simple mannequin" simulation using low cost equipment forms a vital part of that training.

The effectiveness of training clinical skills in a simulator has been described as early as 1969 [1] and the feasibility of such training has been documented in a variety of fields [2]. In-hospital scenario based learning is well established [3]. The aim of this paper is to describe a simple method of training which can be easily integrated into the daily routine of a pre-hospital service, without prohibitive cost. Previously there has been a focus on high-fidelity simulation in pre-hospital care as documented by Batchelder *et al *[4].

In this paper we focus is on "simple mannequin" simulation with an emphasis on "psychological and environmental fidelity" as a means of pre-hospital training. We describe the preparation, scenario design and process of simulation as well as suggestions to maximize the effectiveness of training.

### Definition of "Simple Mannequin" Simulation

"Simple" mannequin simulation refers to the use of mannequins without features like advanced vital signs or programmed response simulation (commonly referred to as high fidelity simulators). Commonly available simple mannequin models include the Laerdal Resuscitation Anne, Ultimate Hurt, Crash Kelly and AmbuMan. Requirements for a "simple mannequin" are shown in Additional file 1.

## Background

The London Helicopter Emergency Medical Service (HEMS) provides a doctor/paramedic team response to major trauma patients in an urban area. The population covered is up to ten million and the service has attended over 21,000 calls since its inception in 1988. Callouts are specifically targeted to patients suffering major trauma via specific despatch criteria and the service aims to provide a large range of critical care skills to patients as early as possible following their injury. The scope of pre-hospital management includes extrication, advanced splinting and haemorrhage control, anaesthesia and sedation and a variety of cardiothoracic procedures including pre-hospital clamshell thoracotomy. Training of staff involves mastering a large body of required reading and equipment and a four week "sign-off" period of mentoring with senior clinicians prior to independent practice. "Simple mannequin" simulation forms a vital part of this training, which we describe below.

### Aims of Simulation

"Simple mannequin" simulation directly facilitates the acquisition and retention of a large range of skills and competencies including but not limited to:

1. Rapid familiarization with equipment and medical packs

2. Enabling practice of critical skills such as rapid sequence intubation

3. Developing crew resource management skills and effective team work between staff

4. Practicing unusual or difficult clinical scenarios such as complicated extrication, multiple patient incidents and unsafe scenes

5. Simulation of rare events such as equipment failure or failed airway protocols

Scenarios are also designed to train and familiarize crews with the equipment that is used less frequently and uncommon but challenging clinical scenarios. They aim to maintain and develop skills and mental preparation for those less common eventualities.

### Method of "Simple Mannequin" Simulation Scenario Development

The scope and number of scenarios are limited only by the imagination of the scenario facilitator but it is important to plan each scenario carefully. All facilitators are experienced pre-hospital care physicians. The initiators for this form of training have all had previous experience with simulation. As the system evolved more facilitators were educated. Most doctors and paramedics who join the service have been involved with facilitating simulation before. This facilitator education takes place within the organisation, and consists of talk-through, formal teaching and then leading scenarios supervised by experienced facilitators. The debrief after a scenario always ends with feedback to the facilitator which ensures continuous development of both the simulation as well as each individual facilitator. Planning begins with setting the particular skills, competencies and events which are to be tested. It is important to limit the focus of each scenario to a few key learning points to provide appropriate emphasis, although some skills such as scene safety assessment, situational awareness, teamwork and crew resource management will be practiced in almost all team based simulations. A realistic mechanism of injury and environmental setting help to maximise immersion in the scenarios for the participants. A mental flowchart of mannequin responses to interventions (or failure to intervene) based on realistic physiology must be developed and used by the scenario facilitator to guide the scenario. If possible it is beneficial to utilize ancillary staff for bystander and external roles and at HEMS London pilots, fire-crew and medical students are routinely used to provide simulated roles such as ambulance crew, police, bystanders and even press. These additional roles provide an important level of realism and test difficult crew resource management issues. There is a great opportunity for bystanders to learn whilst contributing to the training.

### Preparation

**Figure 1:**
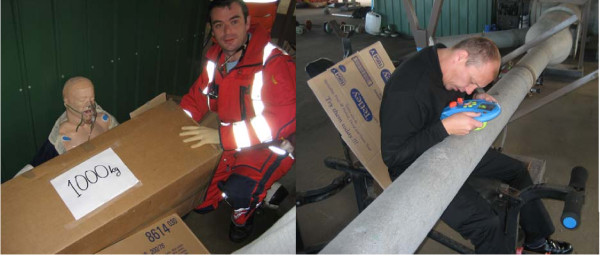
**Local and nearby obstructions are used to create a simulation-scene for i.e. entrapment or road traffic collisions with entrapment**. Using closeby (on the helipad) obstructions makes it feasible to train with the on-call crew. The imagination and creativity of the facilitator and other staff is important for creating a scene that takes the participant into "the zone".

Equipment should be identical to that used in daily operations and prepared and checked as for a real mission. HEMS London uses a clearly marked "training pack" of medical equipment with non-sterile reused disposables to reduce the waste of expensive consumables but which are otherwise indistinguishable from daily operating packs. The mannequin is placed in a position appropriate to the scenario, utilizing realistic obstructions or space limitations. Nearby obstructions can be used to make the scenario scene more realistic for participants (Figure 1). Role playing assistants are briefed on the patient, their injuries and the aims of the scenario and take up their positions. The team is briefed with a realistic "call out" message and pre-hospital mission information as they would on a normal mission. They are then allowed to access the patient.

### Procedure

On arrival at the simulated scene additional briefing is given regarding the scene and age, sex and appearance of the patient. A verbal handover to the HEMS team of the patient's relevant immediate assessment is given by the on-scene role-playing ambulance crew. The scenario facilitator guides the progress of the scenario. Throughout the assessment and management of the patient the scenario facilitator constantly updates the patient status and reports the results of monitoring. Results of interventions are relayed if not immediately simulated on the mannequin. To maintain immersion it is vital that the doctor/paramedic being trained constantly checks the mannequin and monitor for assessment of vital signs and response rather than directly conversing with the scenario facilitator. At the conclusion of the scenario a simulated verbal handover to the receiving hospital is assessed.

### "Rules of the Game"

• All assessments should refer to the mannequin rather than conversing with the scenario facilitator. For example assessment of breath sounds should involve simulated auscultation with a stethoscope with the results announced by the scenario facilitator rather than asking the scenario facilitator "what do I hear when I listen to the chest?"

• All procedures should be performed or simulated where possible. This requires the training crew to remove the equipment from the packs and proceed as far as possible into the procedure i.e. iv access means use of tourniquet, tape/securing the access properly and fluid attachment.

• Lapses or errors should be treated in a realistic fashion. For example, failure to adequately secure a simulated intravenous cannula should result in inadvertent removal.

• The scenario facilitator controls the tempo and progress of the scenario to keep the participants in "the Zone" and tailors the scenario to the participants' performance.

### The "Zone"

The "Zone" refers to the psychological state of simulated realism and immersion that is essential for effective pre-hospital trauma training. The aim is for the training team to believe they are treating a real patient and experience a realistic level of stress. It is achieved by the careful choice and construction of the scenario and by effective guidance of the scenario by the scenario facilitator. The scenario facilitator uses the simulated physiological parameters of the mannequin to direct the need for interventions and determines success or failure of such interventions. The facilitator must remain ahead of the scenario and the simulated responses. The importance of maintaining "The Zone" is constantly emphasized to our scenario facilitators. We believe this facilitates effective learning and is a significant focus of all our training scenarios to ensure that trainees "forget" they are treating a mannequin and experience a high level of psychological fidelity.

### Debrief

All scenarios benefit from immediate debriefs which are structured and guided by a check sheet which is based on one used to debrief real training missions (Additional file 2). Debriefing is essential to maximize learning outcomes. It helps identify erroneous decision making or crew resource management issues and to enables the scenario facilitator to reinforce key learning points. Observers, medical students and non-medical participants all take part in the structured debrief, which is lead by the scenario facilitator. After the formal structured debrief of the participants is completed, there is a formal feedback to the scenario facilitator of their running of the scenario. This is important for the development and improvement of future training and for skill development of scenario facilitators.

Additional file 3 summarises key-points for success in implementing this form for simulation in a service.

## Discussion

Simulation is the process of recreating characteristics of the real world [5]. In general pre-hospital simulation can be divided into part-task training, which refers to replication of a single task or part of a complete process and full mission training which attempts to replicate the environment and interactions of a complex process. Simulation mannequins can be differentiated along a spectrum of fidelity related to the complexity of vital signs simulation or interaction. Fidelity has been characterized by Rehmann *et al *[6] as consisting of three inter-related dimensions. The first dimension, equipment fidelity, refers to the degree to which the simulator replicates the appearance and behaviour of the real system. We believe that it is essential to utilise accurately simulated operational packs and equipment. The second dimension, environmental fidelity refers to the external visual and sensory cues provided by the simulator. The third and arguably the most important dimension, psychological fidelity [5] refers to the degree to which the trainees suspend disbelief and enter into the simulated reality of the situation. This is what we refer to as "the Zone". Whilst high fidelity mannequin simulators have become popular for training in anaesthesia, emergency medicine and advanced life support [7] there have been no published studies which demonstrate a direct correlation between fidelity and training effectiveness [5,7]. Risser *et al *[8] describes how team training can reduce the number of behavioural factors leading to clinical errors, which is similar to our philosophy.

Wisborg et al who have founded and initiated the BEST Foundation (Better & Systematic Trauma Care), describes an effective method of simple training of trauma teams in hospitals in Norway [9]. We believe the same principles apply to the use of operational equipment and realistic surroundings. Our method differs in that there is no need for our operational crew to "go offline" and training can be performed while immediately available as part of a daily operational routine. This makes it even more feasible and ensures that the training is not a "one time event" [5].

Effective use of "simple" mannequin simulation requires a greater focus on situational and psychological fidelity requiring careful scenario development and scenario facilitator involvement. This type of training may more effectively focus training on issues such as team interaction, crew resource management and the goals of training, rather than just the technological features of the mannequin being used.

This form of training focuses on most of the factors described in Anesthesia Crisis Resource Management (i.e. awareness, start of treatment, allocation, declaration, leadership and communication) [10]. These are all factors that are core skills for pre-hospital care team members. These skills are very difficult to acquire and demonstrate without practical training.

**Figure 2:**
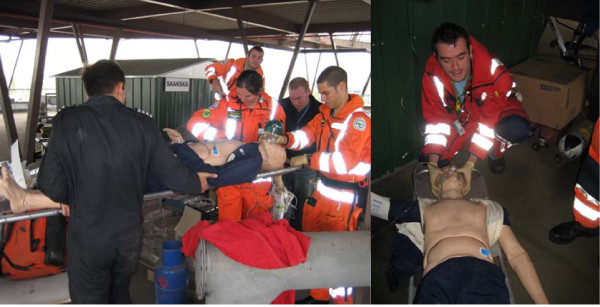
**All members of the team are expected to engage in the scenario training**. It is of importance to participate and act in a realistic manner, use gloves, use stretchers in the same was as in a real-patient situation. Involving other members of staff and using real equipment in the scenario is important for keeping participants in the zone as well as for maintaining a high degree of realism in the scenarios.

We use simulation for training and induction of new staff but also for skills maintenance in established staff. Within the service of London HEMS, There is a high degree of motivation for this kind of training both among paramedics and doctors and indeed motivation among participants is essential to the success of this type of training [11]. This motivation is bolstered by the rotating nature of positions for doctors and paramedics and by making it a compulsory part of training and the daily routines for the on-call crew. We believe that a service implementing this kind of training on a regular basis needs to establish a core of interested people to initiate the programme and from such a foundation seek to involve the whole organization. Figure 2 shows how all members of the team participate and engage in the scenario training.

Simulation is widely used [12] in medical training - particularly in hospital operating room anaesthesia training or teaching specific types of clinical incidents e.g. the management of arrhythmias. However the complexity, cost and fragility of high fidelity and complex mannequin devices limits their utility in realistic outdoor pre-hospital settings. Recently Lee *et al. *have shown that there is no difference between using high- and low-fidelity mannequins for testing of critical care skills [13].

Advantages of "Simple" Mannequin Simulation:

• Reasonable and accessible solution for any organization

• Less risk of damage to the simulator in adverse weather, enclosed spaces and difficult extrication scenarios

• Minimal set-up time and rapid turn-around

• Available for use in any location away from power supplies

• Focuses simulation on the goals of training

## Conclusion

Our experience is that "simple" mannequin training focused on realistic environments and psychological fidelity provides an effective training tool for development of skills in pre-hospital trauma care. It could be rapidly implemented in most services with little expense and minimal disruption to clinical duties. It can be utilized in the daily routine of operational staff and has become an essential part of HEMS London training in the challenging area of pre-hospital trauma care. We hope that the description of our training model will encourage other services to implement similar training at their institutions (Additional file 3) and that studies of the effectiveness of this type of training will follow.

## Competing interests

The authors declare that they have no competing interests.

## Authors' contributions

PB and KH devolved the simulation method and drafted the manuscript, GG developed the simulation system, DL and GD contributed in the writing process. All authors read and approved the manuscript.

## Supplementary Material

Additional file 1 Requirements for a "simple mannequin". Click here for file

Additional file 2 The checklist used for structured debrief after all scenarios. Click here for file

Additional file 3 These are key-points for successfully implement and use simulationtraining in a pre-hospital care service. Click here for file
