# Supplementary material for: Scenario based outdoor simulation in pre-hospital trauma care using a simple mannequin model
Source: Scand J Trauma Resusc Emerg Med. 2010 Mar 15;18:13. doi: 10.1186/1757-7241-18-13 (PMC2845090; doi:10.1186/1757-7241-18-13)
Supplement: Additional file 1 — Requirements for a "simple mannequin". [file 1757-7241-18-13-S1.DOC]

- Realistic size, weight and body articulations
- Free from wires and leads and a requirement for electricity supply or compressor
- Robust, water resistant and washable – may be used in confined spaces and exposed to mud, oil and external environments
- Appropriate anatomical features allowing tracheal intubation to take place
- Readily available for training
