# Supplementary material for: Scenario based outdoor simulation in pre-hospital trauma care using a simple mannequin model
Source: Scand J Trauma Resusc Emerg Med. 2010 Mar 15;18:13. doi: 10.1186/1757-7241-18-13 (PMC2845090; doi:10.1186/1757-7241-18-13)
Supplement: Additional file 2 — The checklist used for structured debrief after all scenarios. [file 1757-7241-18-13-S2.PDF]

Moulage Assessment Sheet

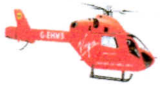

LONDON'S AIR AMBULANCE
roadside intensive care

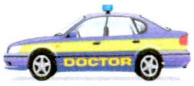

Moulage leader & Assessor	
Doctor	
Paramedic	
Other participants	

Scenario No:

Scoring	Score / 5
1	Vital error (s)
2	Multiple minor error (s)
3	Few minor error (s)
4	Single error
5	Faultless

Scene Management	Score / 5	Examples of Deficiencies
Anticipation		
Simultaneous activity		
Clinical care		
Pace		
Interventions		
Skill tailoring		
Rescue plan		

CRM	Score / 5
Leadership	
Instructions	
Feedback	
Body language	
Language	
Delegation	

Handover or Blue Call	Score / 5
Structure	
Accuracy	
Immediate needs	

Scores	
Global (/5)	
Section Totals	
Total	
