# Supplementary material for: Scenario based outdoor simulation in pre-hospital trauma care using a simple mannequin model
Source: Scand J Trauma Resusc Emerg Med. 2010 Mar 15;18:13. doi: 10.1186/1757-7241-18-13 (PMC2845090; doi:10.1186/1757-7241-18-13)
Supplement: Additional file 3 — These are key-points for successfully implement and use simulationtraining in a pre-hospital care service. [file 1757-7241-18-13-S3.DOC]

**Keys to success**

- Train with accurate medical packs and equipment
- Utilize realistic environments and weather conditions
- Use “bystanders” and actors to simulate on scene personnel (such as police and ambulance service)
- Carefully select the cases and tailor them to the team, their level and learning objectives
- The scenario facilitator must guide the team into “the zone”, to induce realistic time-pressure and stress
- Make sure hospital notification and handover are included in the training
- Give a structured debrief after the scenario
- Use simulation as a regular training form for the operational online crew
